# Gas-Binding Studies of Class 1 Sugar Beet Phytoglobin and C86A Mutant Using Isothermal Spectral Shifts in High-Precision Microliter Assay

**DOI:** 10.3390/ijms26178240

**Published:** 2025-08-25

**Authors:** Leonard Groth, Leif Bülow

**Affiliations:** Division of Pure and Applied Biochemistry, Department of Chemistry, Lund University, 221 00 Lund, Sweden; leif.bulow@tbiokem.lth.se

**Keywords:** phytoglobin, gas-binding assay, microscale fluorescence, ligand binding, nitric oxide, carbon monoxide, protein-ligand interactions, hexacoordinated heme, spectral shift assay, Tween20

## Abstract

Phytoglobins (Pgbs) are plant hemoglobin-like proteins with key roles in nitric oxide (NO) scavenging, oxygen sensing, and hypoxic stress responses. Their typical hexacoordination results in unusually high affinities for gaseous ligands such as NO and carbon monoxide (CO), complicating measurement using conventional methods. Standard assays often require large sample volumes and lack sensitivity for high-affinity, low-abundance proteins like hexacoordinated Pgbs. Here, we present a microscale capillary-based fluorescence assay for the high-precision measurement of protein–gas binding. Fluorophore-labeled proteins are loaded into gas-saturated capillaries and analyzed via dual-wavelength fluorescence to monitor isothermal spectral shifts upon ligand binding. Phosphate-buffered saline with Tween20 (PBS-T20) ensures gas stability and minimizes nonspecific adsorption. Using this approach, we characterized CO and NO binding to the recombinant wildtype (rWT) of *Beta vulgaris* Pgb 1.2 (BvPgb 1.2) and its C86A mutant. CO titrations revealed biphasic binding, with EC_50_ ~400 nM and ~700 μM (rWT) and ~500 nM and ~400 μM (C86A). NO binding showed K_D_ values of ~1600 nM (rWT) and ~400 nM (C86A), implicating Cys86 in ligand affinity. This assay provides a robust, low-volume method for high-affinity protein–gas studies and shows biphasic dynamics in BvPgbs.

## 1. Introduction

Phytoglobins (Pgbs) are specialized hemoglobin-like proteins found exclusively in plants, where they are involved in key processes such as nitric oxide (NO) scavenging and regulation, oxygen (O_2_) sensing, and adaptation to hypoxic conditions [1,2,3,4,5]. In contrast to their animal counterparts, Pgbs predominantly adopt a hexacoordinated configuration, which results in exceptionally strong binding to gaseous ligands like NO [5] and carbon monoxide (CO) [6,7,8]. These distinctive ligand-binding properties make it difficult to determine their binding affinities through standard analytical approaches [9,10]. Assaying binding affinities between proteins and gaseous species in solution, such as CO and NO, is a fundamental task in biochemistry, with implications for understanding enzymatic catalysis, signaling pathways, and protein functionality [11,12,13]. Similarly important are a range of biophysical characterizations of proteins in these ligand-bound states. These gaseous molecules are critical in a variety of biological processes [14,15,16,17], highlighting the need for precise measurement of their interactions with proteins [18]. However, traditional experimental setups for such studies often present practical and technical challenges, especially for high-affinity Pgbs, which can have dissociation constants (K_D_) ≪ 1 μM [9].

The microscale capillary-based technology described here employs isothermal spectral shift (SpS). SpS has previously primarily been used for the determination of protein–protein interactions [19,20]. In this study, we examine the possibility to extend the use of this technique to also include the binding of small ligands, like gases, to proteins. Ligand binding induces shifts in fluorescence spectra (blue- or red-shifts), which are detectable under isothermal conditions via dual-wavelength fluorescence detection at 650 nm and 670 nm. By plotting fluorescence intensity ratios (670/650 nm) against ligand concentration, the SpS-derived K_D_ can be obtained [20]. Our goal is to determine K_D_ or half maximal effective concentrations (EC_50_) from ligand titration series. Here, we present a streamlined microscale method employing fluorophore-labeled proteins in gas-saturated microscale capillaries. The assay significantly reduces the amounts of protein required and experimental complexity, providing efficient and accurate binding affinity measurements.

## 2. Results

### 2.1. Initial Protein Modifications

**Figure 1:**
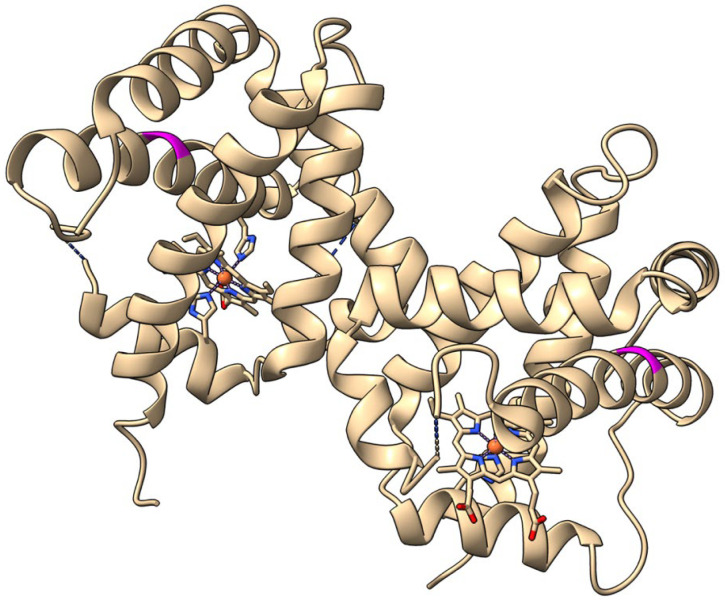
Image of dimeric Beta vulgaris class 1 phytoglobin (BvPgb 1.2) cysteine-to-alanine substituted mutant (C86A, PDB ID: 7Z1U) with the site of substitution colored in magenta.

The two Pgbs examined, BvPgb 1.2 (PDB: 7ZOS) and its alanine-substituted mutant (C86A) (PDB: 7Z1U) (see Figure 1), could be purified from recombinant *E. coli* cells according to previously developed protocols [21]. By using a two-step chromatographic procedure, the proteins were obtained in a highly purified form. The proteins were subsequently covalently labeled at primary amines (i.e., N-termini and lysine residues), respectively, utilizing a dye carrying a reactive NHS-ester group. After removal of excess labeling reagents, the proteins could be used directly for gas binding studies.

The proteins were thus incubated in solutions enriched with either CO or dissolved NONOate, utilizing a loading buffer (PBS-T20) to maintain gas saturation. Subsequent titrations of gas-enriched solutions yielded clear, reproducible dose–response curves.

These findings reveal intriguing functional properties of BvPgbs, proteins known to form dimers. The observation of two distinct transition events in the dose–response curves may indicate cooperativity in ligand-binding behavior, an effect documented in other globins [22] but previously unreported in BvPgbs. However, previous studies on Pgbs suggest that biphasic or multiphasic kinetics typically arise from the existence of multiple conformational states [23,24,25,26], each exhibiting distinct ligand-binding characteristics. Unlike pentacoordinate globins, hexacoordinate globins thus exist in multiple conformational states. Trent et al. model their reaction scheme in [27] as (1) a closed hexacoordinated state, (2) an open hexacoordinated state, (3) an open pentacoordinated state, and finally, (4) the ligand-bound form. The transition from the closed to open state is slow and not in rapid equilibrium, meaning that traditional kinetic assays, such as stopped-flow rapid mixing and flash photolysis, are unable to fully resolve the multiphasic binding behavior typical of these states [27,28,29].

All SpS measurements conducted in this study were performed using the Monolith X instrument (NanoTemper Technologies GmbH, München, Germany), which is equipped with dual-emission detection optics. The MO.Control 2software (NanoTemper Technologies GmbH) was used to calculate fluorescence intensity ratios at 670 and 650 nm. K_D_ values were determined by fitting dose–response curves to a binding model that follows the law of mass action with a 1:1 interaction stoichiometry. This model defines the bound fraction, *f*(*c_L_*), at equilibrium for a given ligand concentration, *c_L_*, as given by Equation (1):

(1) $$f\left(c_{L}\right)=\frac{c_{L}+c_{T}+K_{D}-\sqrt{{\left(c_{L}+c_{T}+K_{D}\right)}^{2}-4c_{L}c_{T}}}{2c_{T}}$$

where *c_T_* is the final concentration of target in the assay. The *K_D_* is estimated by fitting Equation (1) with the ratiometric expression seen in Equation (2):

(2) $$R\left(c_{L}\right)=\frac{R_{f}+f\left(c_{L}\right)\cdot \left(R_{b}-\frac{F_{b}}{F_{f}}\cdot R_{f}\right)}{1+f\left(c_{L}\right)\cdot \left(\frac{F_{b}}{F_{f}}-1\right)}$$

where *R*(*c_L_*) represents the 670 nm/650 nm ratio at a given *c_L_*, *R_f_* corresponds to the ratio of the free target, and *R_b_* corresponds to the bound complex. The parameters *F_b_* and *F_f_*, derived from the sigmoidal dose–response curve fitting of the free target and bound complex, respectively, adjust for ligand-induced changes in fluorescence intensity at 650 nm [20].

### 2.2. CO Measurements

**Figure 2:**
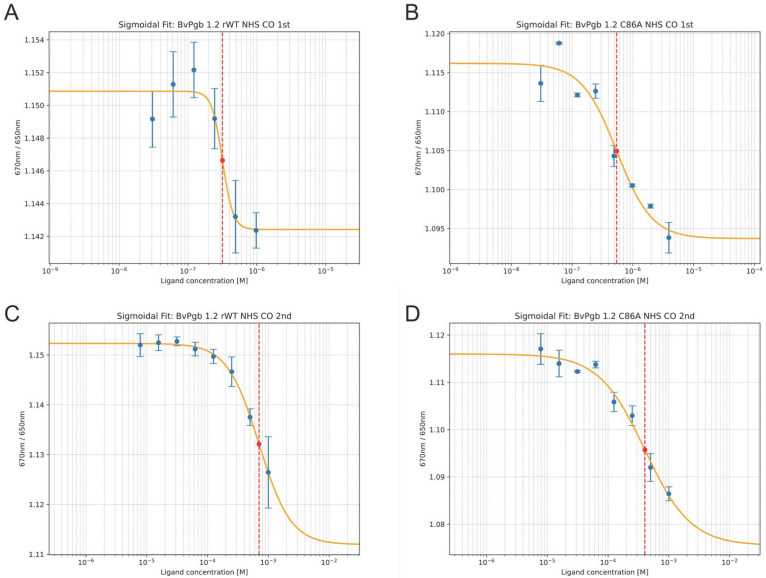
Carbon monoxide (CO) affinity to BvPgb 1.2 recombinant wild type (rWT) (left) and C86A (right) can be effectively characterized using isothermal spectral shift. Two transitions or dose–response curves were clearly observed for BvPgb 1.2 rWT, with transitions and corresponding half maximal effective concentrations (EC_50_) for the rWT highlighted in (**A**) at ~400 nM and (**C**) ~700 μM for the first and second transition, respectively, and similarly at ~500 nM and ~400 μM for the C86A in (**B**,**D**).

Titration of CO revealed two clear dose–response curves at different intervals independent of the cysteine residue, namely within the nM and the µM range, respectively (see Figure 2). For CO binding, the effective ligand concentrations (EC_50_) required to achieve a 50% response were approximately 400 nM in the first transition and 700 μM in the second transition for rWT. The C86A mutant exhibited EC_50_ values of approximately 500 nM in the first transition and 400 μM in the second transition.

### 2.3. NO Measurements

**Figure 3:**
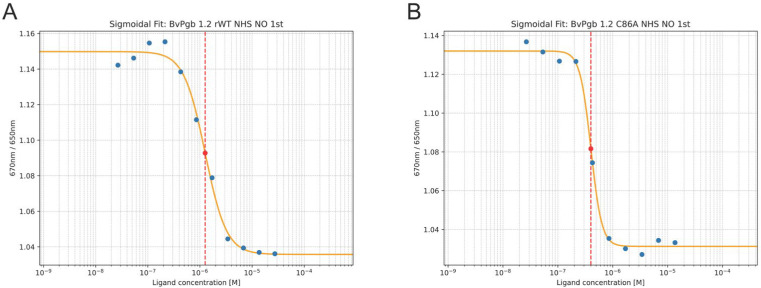
Nitric oxide (NO) affinity to BvPgb 1.2 rWT (**A**) and C86A (**B**) can be effectively characterized using isothermal spectral shift. Two transitions or dose–response curves were clearly observed for BvPgb 1.2 rWT, with transitions and corresponding dissociation constants (K_D_) for the rWT highlighted in (**A**) at 1600 nM and at 400 nM for C86A in (**B**).

Biphasic kinetics was not readily observed in the NO titration case. A clear dose–response curve was nevertheless observed for both the rWT and C86A mutant (see Figure 3), with the C86A mutant having a K_D_ at 400 nM, almost a quarter of the K_D_ of rWT at 1600 nM.

### 2.4. Comparison with Prior Binding Data

Our obtained results are consistent with K_D_ values derivable from earlier work [30], where kinetic parameters for BvPgb 1.2 were obtained using stopped-flow rapid mixing and flash photolysis, allowing the calculation of a single apparent *K_D_* per gas. While not explicitly reported, *K_D_* can be estimated from those data using Equation (3):

(3) $$K_{D,obs}=\frac{k_{off}(1+K_{H})}{k_{on}}\;\;\;$$

where *K_H_* is the hexacoordination equilibrium constant and the (1 + *K_H_*) factor is used to account for the “penalty” imposed by the hexacoordinated fraction. Using Equation (3) on data from [30], we estimate K_D_ (CO) ≈ 100 μM and K_D_ (NO) ≈ 6400 nM for BvHb 1.2, values that align well with the affinities measured in our current study for the higher-affinity state (400–700 μM for CO and 400–1600 nM for NO). The additional lower K_D_ or EC_50_ for CO (400–500 nM) observed with our method likely reflects the contribution of the closed protein form, consistent with the 100–1000-fold kinetic differences reported in [27,28,29].

The results from our SpS measurements are compiled in Table 1 together with previously obtained binding data employing flash photolysis and stopped-flow rapid mixing [30].

The 16-point SpS assay consumed a total of 3.2 nanomoles of Pgb, representing a more than 30-fold reduction in sample consumption compared to the 100 nanomoles required for the six- and four-point datasets generated by stopped-flow rapid mixing and flash photolysis assays, respectively, each performed with 1 mL of 10 µM protein [30].

## 3. Discussion

This study presents a microscale assay tailored for gas-binding studies, offering a technologically simple and rapid alternative to conventionally used methods. Using capillary-based SpS measurements, we establish a versatile and sensitive platform that requires only microliter-scale sample volumes, representing an important advancement when studying proteins that are difficult to express or purify in large quantities, such as Pgbs. Pgbs are increasingly recognized for their roles in NO scavenging, plant physiology, and stress responses [7,14,31], yet have remained underexplored partly due to technical barriers associated with their gas-binding characterization [9]. Our study addresses this limitation by presenting one of the first successful adaptations of the SpS technology for quantitative analysis of protein–gas interactions, thus expanding its utility beyond traditional ligand–protein or protein–protein binding studies. SpS is based on changes in protein optical properties, specifically absorbance or intrinsic fluorescence, upon ligand binding [20].

This approach circumvents the need for continuous gas flow, which can be cumbersome and require specialized setups, especially so if the gases are hazardous like CO. Instead, SpS assays enable precise thermodynamic data collection from equilibrium conditions, offering a more accessible and scalable alternative to conventional gas-binding methods such as stopped-flow spectroscopy or flash photolysis, where sample consumption is a recognized problem [32]. In stopped-flow rapid mixing, setups exceeding 3 mL and subsequent experimental runs at 1 mL are not uncommon [33]. The ability to decouple the experimental setup from specialized gas-dosing instrumentation and reduce sample consumption more than 10-fold significantly lowers the barrier to entry for labs seeking to study gas-binding proteins, particularly in resource-limited settings.

Moreover, conventional kinetic assays are unable to capture certain types of reactions. Stopped-flow rapid mixing, for example, can only monitor reactions that are longer than the instrumental dead-time. Whereas flash photolysis can observe short-lived reactions [34], these methods report ensemble-averaged kinetics and generally cannot resolve multiple conformational substates unless they produce distinguishable spectroscopic signals [35]. Neither of these issues are present in the current SpS assay, which determines K_D_s at equilibrium [20].

To further probe the regulatory features of gas binding, we investigated the role of C86 through site-directed mutagenesis. The C86A mutant maintained the ability to bind both CO and NO but exhibited altered EC_50_ values compared to rWT, particularly for NO. This result indicates that while C86 is not directly involved in heme–ligand coordination, it plays a modulatory role, by influencing the tertiary or quaternary structure of the protein [21,36].

Our NO-binding data also align with the established function of Pgbs in the NO–Pgb cycle, where these proteins act as NO scavengers, converting NO to nitrate via oxidation reactions involving O_2_-bound heme [37,38]. Under hypoxic stress, this function helps to regulate intracellular NO levels, contributing to plant survival and adaptive signaling [8,10,39,40]. By revealing a potential redox-sensitive tuning mechanism via C86, our results add a new layer of regulatory nuance to this pathway and may help to explain how Pgbs maintain functionality across fluctuating environmental conditions.

Beyond plant systems, the versatility of this assay suggests broad applicability to a wide range of gas-binding proteins from various biological domains. Potential applications include the study of microbial gas sensors (e.g., H-NOX domains), mammalian hemoproteins, or engineered gas-binding proteins used in synthetic biology.

From a methodological standpoint, the assay’s minimal material requirements and adaptability mark an improvement over traditional gas-binding methods. It is particularly well suited for pipeline research, which requires rapid data acquisition, cost efficiency, and the capacity to work with novel or low-abundance proteins [41]. Moreover, the adaptability of the assay to different gases, redox conditions, and surfactant systems provides a valuable framework for future optimization and customization based on experimental needs.

## 4. Materials and Methods

All of the water used had a resistivity of 18.2 MΩ·cm and a total organic content of less than five parts per billion. All spectrophotometric measurements were performed on a Cary 60 UV/Vis system (Agilent Technologies, Santa Clara, CA, USA), unless otherwise stated. All primers were purchased from Integrated DNA Technologies, Inc. (IDT, Coralville, IA, USA) as single-stranded DNA oligos (100 μM) resuspended in 10 mM Tris, 0.1 mM EDTA (pH 8.0) after purification via standard desalting.

### 4.1. Protein Expression and Purification

BL21-DE3 *Escherichia coli* were transformed to express the rWT and the C86A mutant as described previously [21]. Briefly, transformed cells were cultivated in terrific broth (TB) medium supplemented with 100 µg/mL carbenicillin at 37 °C and 150 rpm until OD_600_ ≥ 2, whereupon cells were induced by the addition of 0.5 mM IPTG and 0.3 mM δ-aminolevulinic acid. To stabilize and inhibit the potentially deleterious activity of nascent Pgbs, the medium was sparged with CO and shake flasks were sealed to maintain a CO-saturated environment during expression. Induced cells were incubated in the dark at 22 °C and 150 rpm for 16 h to promote proper holoprotein synthesis while minimizing proteolysis, aggregation, and heme photodegradation.

Cells were then pelleted and resuspended in 50 mM Tris–HCl, pH 8.5 (2 mL/g cells), and lysed with a Maximator^®^ High Pressure Homogenizer. Lysate was clarified by means of centrifugation at 11,000 rpm for 20 min at 4 °C before being dialyzed for 36–48 h against the same buffer using a 6–8 kDa cutoff membrane, refreshing the buffer once after 24 h.

Dialyzed lysate was purified through strong anion exchange chromatography, followed by a hydrophobic interaction polish. First, proteins were loaded onto a Q-Sepharose FastFlow (QFF) column equilibrated with 50 mM Tris–HCl, pH 8.5, on an ÄKTA™ Avant system. Elution occurred in two steps: first, 1 CV with 50 mM Tris–HCl, 200 mM NaCl, pH 8.5 (conductivity ~4.0 mS/cm), followed by 9 CVs to reach ~11.0 mS/cm.

Fractions with a high heme-protein content, as identified by absorbance at the Soret peak from the anion exchange, were pooled and then supplemented with 1 M ammonium sulfate (NH_4_)_2_SO_4_ by means of the gradual dropwise addition of saturated (NH_4_)_2_SO_4_ solution. The salted-out protein solution was then mixed for 30 min in the dark, clarified by centrifugation, and filter-sterilized (0.45 µm) before polishing. Polishing was performed on a Butyl-Sepharose HP (BHP) column pre-equilibrated with 50 mM Tris, 1.5 M (NH4)_2_SO_4_, pH 8.5. The proteins were eluted using a 10 CV linear gradient from 1.5 to 0 M (NH4)_2_SO_4_. Fractions were again pooled based on Soret peak absorbance reads and then concentrated with 10 kDa MWCO Viva-Spin columns (Sartorius, Göttingen, Germany). Concentrated proteins were flash-frozen with liquid nitrogen (LN_2_) and stored at −80 °C for down-stream use.

The protein content of dialyzed cell lysate, QFF purified protein solution, BHP polished protein solution, and the final concentrate was measured via the Bradford assay. Purity was similarly assessed using SDS-PAGE.

### 4.2. Protein Labeling and Capillary Loading

Covalent labeling of primary amines was performed using the Protein Labeling Kit RED-NHS 2nd Generation (cat# MO-L011; NanoTemper Technologies GmbH). The assay buffer (PBS-T20) was prepared by supplementing degassed PBS pH 7.4 (cat# 21-040-CV; Corning, NY, USA) with 0.05% Tween20. Purified proteins in Tris were subjected to buffer exchange using the A-column prior to labeling. Briefly, a twofold molar excess of dye was added to the protein prepared to a final concentration of 20 µM. Proteins and dye were incubated for 30 min at room temperature in the dark to avoid photobleaching. Free dye was removed from the labeled protein using the provided B-column. Labeled proteins were kept in the dark and on ice awaiting capillary loading.

A working 1 mM CO solution was prepared by sparging PBS-T20 with CO, with the concentration approximated through Henry’s law for CO dissolvability in water (k_H_(CO) ≈ 0.001 mol l^−1^ bar^−1^). A working 2 mM NO solution (k_H_(NO) ≈ 0.002 mol l^−1^ bar^−1^) [42] was prepared by dissolving 25 mg of NONOate (cat# D184-25MG; Merck, Darmstadt, Germany) in 15 mL of PBS-T20 to obtain a 10.75 mM solution, which provides a 10-molar excess of saturation upon decomposition. The NONOate PBS-T20 solution was gently agitated to create a gas-buffer zone to capture decomposing NONOate. Ligand solutions were prepared as 16-point serial dilutions of 10 µL in PCR-strip tubes, before being mixed by means of aspiration with 10 µL of labeled protein and incubated for 5 min. Complete assay samples were then loaded onto premium capillaries (Cat# MO-K025; NanoTemper Technologies GmbH), sealed with Capillary Sealing Paste (cat# PR-P001; NanoTemper Technologies GmbH) to prevent evaporation, and placed into the device.

### 4.3. Dual-Emission Spectral Shift Measurements

All SpS measurements in this work were performed using the Monolith X instrument equipped with dual-emission detection optics. All measurements were performed in triplicate. CO measurements were replicated four times for rWT and twice for C86A.

After fitting, the standard deviation (*σ_KD_*) of log_10_(*K_D_*) was extracted as the square root of the relevant diagonal element of the covariance matrix. The uncertainty for K_D_ was then propagated as following standard approaches for parameter uncertainty estimation and error propagation in nonlinear regression [43].

$$\sigma_{KD}=ln\left(10\right){\cdot K}_{D}{\cdot \sigma }_{\log_{10}\left(K_{D}\right)}$$

## 5. Conclusions

By combining microscale precision with practical accessibility and methodological innovation, we present an assay system that expands the toolkit for investigating gas–protein interactions. Our findings demonstrate the utility of capillary-based SpS for capturing K_D_s due to subtle conformational effects arising from gaseous ligand binding. This study supports previous models on biphasic ligand behavior in hexacoordinated Pgbs. It also investigates the role of C86 as a potential redox switch. Additionally, this work opens new avenues for gas biology research in both fundamental and applied contexts.

## Figures and Tables

**Table 1:** Comparison of K_D_s as measured through isothermal spectral shift and traditional kinetic assays of flash photolysis and stopped-flow rapid mixing from previous research [30] on BvPgb 1.2.

	Isothermal Spectral Shift ^1^			Flash Photolysis and Stopped-Flow Rapid Mixing ^2^		
	K_D(NO)_	K_D(CO, open)_ /EC_50,1_	K_D(CO, closed)_ /EC_50,2_	K_D(NO)_	K_D(CO, open)_	K_D(CO, closed)_
Protein	[>nM]	[μM]	[nM]	[nM]	[μM]	[nM]
rWT	1600 ± 100	700 ± 200	400 ± 100	~6400	~100	N/D
C86A	400 ± 100	400 ± 300	500 ± 200	N/D	N/D	N/D

^1^ As measured in this work. ^2^ As calculated per Equation (3) using kinetic parameters measured and reported in [30].

## Data Availability

Data supporting the findings of this study are available upon request from the corresponding author.

## Abbreviations

The following abbreviations are used in this manuscript:

BHP	Butyl-sepharose HP, used in aliphatic hydrophobic interaction chromatography
BvPgb 1.2	Class 1 phytoglobin from Beta vulgaris
C86A	Mutant of BvPgb 1.2 with cysteine-to-alanine substitution at position 86
CV	Column volume
EC50	Half maximal effective concentration
KD	Dissociation constant
KH	Hexacoordination equilibrium constant
kH	Henry’s law solubility constant
LN2	Liquid nitrogen
N/D	Not determined
NHS	N-Hydroxysuccinimide
PBS-T20	Phosphate-buffered saline supplemented with 0.05% Tween20
Pgb	Phytoglobin
QFF	Q-Sepharose FastFlow, used in anionic exchange chromatography
rWT	Recombinant wild type of BvPgb 1.2.
SpS	Spectral shift
TB	Terrific broth
